# An Application of Fuzzy Analytic Hierarchy Process in Risk Evaluation Model

**DOI:** 10.3389/fpsyg.2021.715003

**Published:** 2021-09-22

**Authors:** Geng Peng, Lu Han, Zeyan Liu, Yanyang Guo, Junai Yan, Xinyu Jia

**Affiliations:** ^1^Business School, Jiangsu University of Technology, Changzhou, China; ^2^School of Economic and Management, Tongji University, Shanghai, China; ^3^Business School, University of International Business and Economics, Beijing, China; ^4^China Ordnance Materials Huabei Co., Ltd., Taiyuan, China; ^5^School of Management Science and Engineering, Shanxi University of Finance and Economics, Taiyuan, China; ^6^Department of Electrical Engineering, Tsinghua University, Beijing, China

**Keywords:** land expropriation, government governance, risk assessment, mass unexpected, evaluation methods

## Abstract

Conflicts in land exploration are incisive social problems which have been the subject in many studies. Risk assessment of land conflicts is effective to resolve such problems. Specifically, fuzzy mathematics and the analytic hierarchy process were combined together to evaluate risk in land conflicts in our work, which is proved useful to solve uncertainty and imprecision problems. Based on the analysis of the principles for the risk assessment of a land conflicts index system, a set of risk assessment indexes using the fuzzy analytic hierarchy process (FAHP) was presented. The results show that the overall risk is at medium level, and the risk of the feasibility index and controllability index need to be paid more attention to. The contribution of this article is reflected in two aspects: (1) the application of FAHP in risk assessments of land conflicts is effective and valid; (2) it is helpful for governments to establish a stricter management system of work safety for conflicts in land exploration based on the risk assessment results.

## Introduction

With the development of urbanization, there have been violent conflicts caused by land expropriation which have caused significant casualties and economic losses (Bao et al., 2019). The conflicts in land expropriation are major problems during urbanization in developing countries (Tagliarino et al., 2018), which not only seriously hinder the development of urbanization, but also cause sharp social contradictions which have seriously harmed social stability and development (Mathur, 2013). Conflicts in land expropriation have already become key problems affecting social stability.

The damage of conflicts in land expropriation has received a lot of interest and considerable theoretical analysis (Cao et al., 2018). Land expropriation is closely related with farmers’ vital interests, involving the larger population as they widely and seriously affect the stability of social society (Li and Xi, 2019). Conflicts in land expropriation are always regarded as verbal confrontation, vandalism, expulsion, physical altercations, and, at the extreme, assassinations and so on (Hui and Bao, 2013). Causes of conflicts in land expropriation have been studied which mainly refer to institutional flaws, illegal land acquisition by local governments, and disharmony between the rapid expansion of the city and space conversion. Some scholars proposed that: conflicts in land expropriation reflect the deep hidden contradiction between urban planning and economic development (Ma et al., 2020). Its essence is the interest conflict of the stakeholders (He and Asami, 2014). However, the causes of conflicts in land expropriation are complex. Different regions have different causes of conflicts in land expropriation. How to prevent conflicts in land expropriation has become a top priority. In order to prevent conflicts in land expropriation, risk assessment of conflicts in expropriation is an effective way. There are lots of risk assessment methods for multi-criteria decision-making in uncertain environments, such as the alpha-discounting method (Smarandache, 2010, 2013a,b); randomness and fuzzy theory (Abdo and Flaus, 2016); evidential reasoning algorithm and fuzzy set theory (Chen et al., 2014); Markov chain Monte Carlo simulation (Faghih-Roohi et al., 2014); the improved analytic hierarchy process (AHP) (Li et al., 2013); and variable fuzzy sets model and fuzzy AHP (Zou et al., 2013).

Although there are lots of risk assessment models applied to different risk assessment projects, fuzzy analytic hierarchy process (FAHP) especially has a wide application in many decision-making environments. However, the application of FAHP in the risk assessment of conflicts in land expropriation is not sufficient. The research gap shows that causes of conflict in land expropriation are extremely complex which are different in different regions, therefore it is necessary to identify specific risk factors systemically according to local conditions using FAHP. The motivation of this article is to find an effective risk assessment method which can prevent conflicts in land expropriation. Specifically, fuzzy mathematics and the AHP are combined together to assess risks in land conflicts, the results of risk assessment can reflect the total risk degree of land expropriation and some specific aspects which mostly lead to emergency.

In what follows, we first conduct a literature review of “Risk Causing Factors of Conflicts in Land Expropriation” and “Risk Assessment Model of Conflicts in Land Expropriation” given in section “Literature Review.” Subsequently, we will introduce research materials and methods in section “Materials and Methods.” Then, we choose one typical land expropriation program as a case study to analyze in section “Case Study.” Finally, the discussion and future prospects are presented in section “Discussion”.

## Literature Review

It is challenging to establish a risk assessment model for conflicts in land expropriation with decision-making in uncertain environments. There are several methodologies for risk assessment: the AHP (Saaty, 1977); Gray system theory (Zhou et al., 2015); multi-state Bayesian network methodology (Qiu et al., 2015); and fuzzy mathematics (Wang, 2019). AHP is an effective multi-target decision-making method combining qualitative analysis with quantitative analysis which is often applied in comprehensive evaluation. Traditional AHP is widely used in risk assessment, which has been increasingly improved (Li et al., 2013). The advantage of AHP is that this method supports the weight of qualitative indicators and the rationality of the subjective factors (Feng et al., 2014). However, there are some limits of traditional AHP when we assess risks of conflicts in land expropriation. The result may not be consistent using traditional AHP. Consequently result reliability is not high. The weights of each index reflect relative importance between two factors which is determined according to the judgments from experts. However, the judgments from several experts vary, which may result in a bias in the result evaluated using a single weight index. What is more, the decision maker’s perception is associated with a crisp number using traditional AHP which is not suitable for uncertain data and an ambiguous decision-making environment. The theory of fuzzy mathematics is an effective solution to resolve such a problem in an uncertain environment and has been continuously improved, variations include: the randomness and fuzzy theory (Abdo and Flaus, 2016), evidential reasoning algorithm and fuzzy set theory (Chen et al., 2014), the combination of fuzzy AHP and TOPSIS (Taylan et al., 2014), and variable fuzzy sets model and fuzzy AHP (Zou et al., 2013). The calculation process and theory of fuzzy mathematics is relatively easy to follow in which we can describe the fuzzy character of classified bounds and reflect on the actual situation with objectiveness (Jiang et al., 2009). However, its objective membership functions are difficult to determine and it cannot well solve the information duplication problem caused by related assessment indices (Liu et al., 2010). It is necessary to combine fuzzy mathematics and AHP together to assess risks. The FAHP approach is primarily used for multi-attribute analysis and structured hierarchy decision situations which is better at obtaining priority weight vectors regarding multi-attribute decision-making than AHP (Lee, 2015). FAHP is a practical method for dealing with fuzziness and uncertainty which has been widely used in the risk assessment of different areas especially in assessment for some high-risk projects: risk assessment of earthquake triggers (Lyu et al., 2020a); ecosystem health assessment (Sun et al., 2019); and mega-city infrastructures related to land subsidence (Lyu et al., 2020b). FAHP is an improved method based on traditional AHP with the use of fuzzy numbers which could determine uncertainties in mapping human preferences into a score when considering different criteria in the selection procedure (Hamidi et al., 2010).

Fuzzy comprehensive evaluation (FCE) is a crucial step in the assessment of the risk of conflicts in uncertain environments (Geng et al., 2020). The advantage of FCE is that the contribution of multiple related factors can be comprehensively considered according to the weight. With the development of fuzzy theory, FCE was developed as an effective means of dealing with the interdependence problem of various factors, and has been widely applied in decision-making and assessment processes in imprecise environmental situations. The integration of FAHP with FCE is to overcome the uncertainty embedded in Likert-type variables and avoid subjective evaluations. It not only solves the problem of the fuzzy concept but also combines with FAHP to form a complete set of evaluation systems. However, few studies have applied this method to evaluate risks of conflicts in land expropriation as the core concept. This article applied FAHP-FCE in a risk assessment model of conflicts in land expropriation. The objective of this study is to provide a risk assessment model combined with FAHP and FCE. The FAHP methodology is used to calibrate the weights of risk assessment factors, and the FCA approach is used to assess risks of specific land expropriation projects based on the index system obtained by the interval FAHP from an objective perspective.

## Materials and Methods

### Data Source and Questionnaire Design

A questionnaire is the main means of collecting data in this article. There are two approaches for the experts’ questionnaire: one is pairwise comparison (Saaty, 2003); the second method uses table comparison (Lyu et al., 2020a). The second new questionnaire method cannot only get the appropriate experts’ reply but also can determine the fuzzy number based on experts’ replies. We used the second approach. The specific questionnaire we designed is presented in Appendix. The nine scores represent the relative importance of the contribution of a factor to risk of conflicts in land expropriation (from 1 = lowest importance to 9 = highest importance). It is especially an effective method to collect different viewpoints from numbers of experienced experts for a complex decision-making problem that involves a large number of risk factors.

Specifically, we invited six experienced experts with expertise in land expropriation and emergency management. These experts included two academic professors whose research direction was emergency management especially for conflicts in land expropriation, two government workers who have a rich experience for solving conflicts in land expropriation, and two contractors who have rich experience for land expropriation. Referring to the practice of literature (Lyu et al., 2020a), there are two requirements that need to be satisfied when filling out this questionnaire. Firstly, each expert needs to assign an integer between one and nine to a factor. Secondly, each expert tries to give different scores to different factors in the same layer as much as possible, because the judgment matrix may be useless when all factors are given the same importance in the same layer.

### Triangular Fuzzy Number Design

Triangular fuzzy number is a useful way to solve problems of decision-making in uncertain environments. It is a method of triangular fuzzy number to represent fuzzy comparative judgment. Instead of a crisp number, the triangular fuzzy number is more suitable for expert judgment whose judgment is ambiguous (the minimal value, the most likely value, and the maximum value). Based on the previous research of triangular fuzzy number (Sun et al., 2019), we denoted triangular fuzzy number as:


(1) P=(l,μ,m)

**FIGURE 1:**
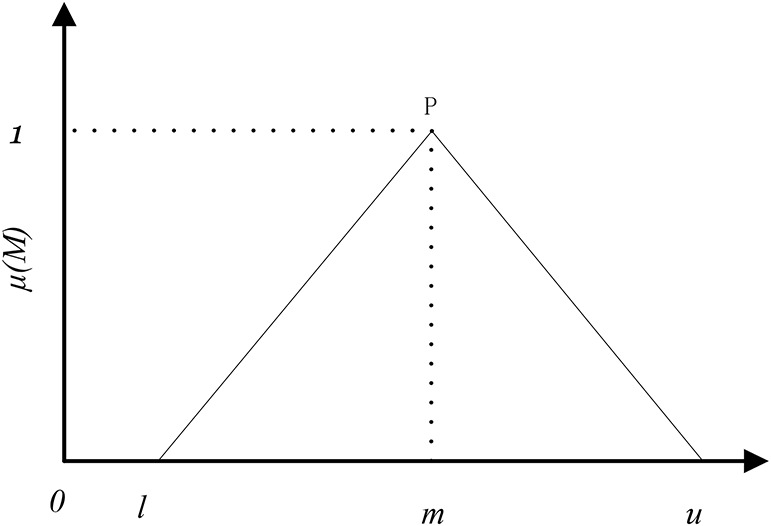
Triangular fuzzy number (P).


Figure 1 shows the membership function of a triangular fuzzy number. Parameter l represents the minimal value, parameter u represents the most likely value, and parameter m represents the maximum value. The membership function of *M*(μ) is defined as Eq. 2.

(2) $$\mu \,\left(x|M\right)=\left\{ \begin{array}{c} \begin{array}{cc} 0 & \left(x<l\right) \end{array}\\ \begin{array}{cc} \frac{x-l}{m-l} & \left(l\leq x<m\right) \end{array}\\ \begin{array}{cc} \frac{\mu -x}{\mu -m} & \left(m\leq x<\mu \right) \end{array}\\ \begin{array}{cc} 0 & \left(x\geq \mu \right) \end{array} \end{array}\right.$$

A judgment matrix *F*_*n×n*_ with the ratio of interval values is written as Eq. 3, where *P*_1_*_*n*_* represents the ratio of the interval value for the first factor and the interval value for the nth factor; and *P*_*n*__1_ represents the reciprocal of *P*_1_*_*n*_*.


(3) $$F_{n\times n}=\left[\begin{array}{ccc} 1 & \text{L} & P_{1\,n}\\ \text{M} & \text{O} & \text{M}\\ P_{n\,1} & \text{L} & 1 \end{array}\right]$$

**FIGURE 2:**
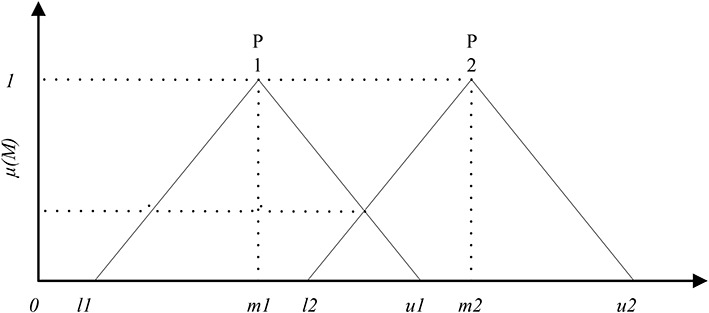
Membership function between triangular numbers P1 and P2 in the fuzzy evaluation matrix.

Based on the results of *P*_*i*_, the membership between triangular numbers *P*1 = (*l*_1_*m*_1_*u*_1_) and *P*1 = (*l*_2_*m*_2_*u*_2_) in the fuzzy evaluation matrix can be plotted as Figure 2.

### The Integration Between Fuzzy Analytic Hierarchy Process and Fuzzy Comprehensive Evaluation

**FIGURE 3:**
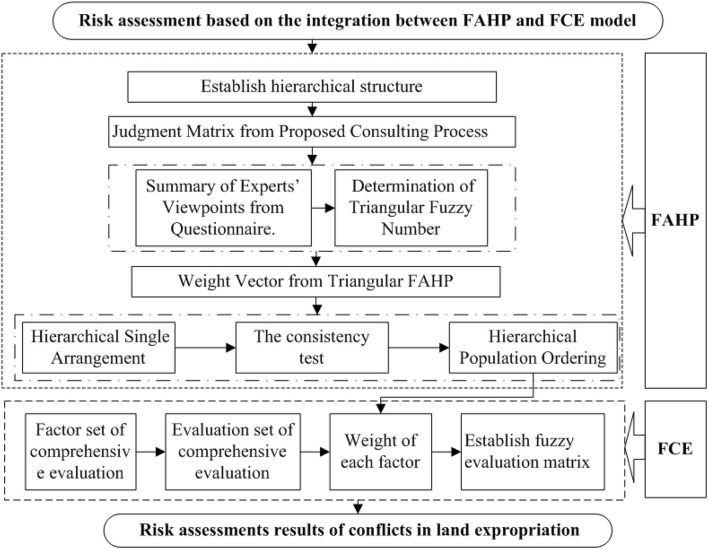
Flow pipe of the risk assessment using FAHP-FCE.

Figure 3 shows the flow pipe assessing the conflicts risk in land expropriation based on FAHP and FCE. The assessment procedure includes two major sections: (1) determining the weight vector of risk factors using FAHP; (2) calculating the risk level of specific land expropriation projects using FCE based on the evaluation index weight using FAHP (Chen et al., 2014).

The procedure can be described in detail as follows: (1) establish hierarchical structure based on the influential factors identified previously; (2) establish judgment matrix from the proposed consulting process, including the summary of experts’ viewpoints from the questionnaire and determination of triangular fuzzy number; (3) calculate weight vector based on triangular FAHP, specific steps including: hierarchical single arrangement; consistency test, and hierarchical population ordering; and (4) calculate the risk level of specific land expropriation projects using FCE based on the evaluation index weight using FAHP. More detailed steps are described in section “Case Study.”

## Case Study

There are several reasons for choosing SY as a case city to research conflicts in land expropriation. Firstly, SY is a typical city which has lots of land expropriation projects. In recent years, there have been many violent conflicts caused by land expropriation. Hundreds of villagers were injured in the conflicts, which caused a bad social impact. The conflicts in land expropriation of SY could represent most of the characteristics of general conflicts in land expropriation. Secondly, the authors provide a long-term consulting service for the local department of government; we have access to data and materials of conflicts in land expropriation of SY. Thirdly, the invited experts are not only professional and have academic knowledge, but are also familiar with local conditions especially management experience and risk points that may cause conflicts in land expropriation.

### Weights of the Assessment Factors Using Fuzzy Analytic Hierarchy Process

#### Hierarchical Structure

**FIGURE 4:**
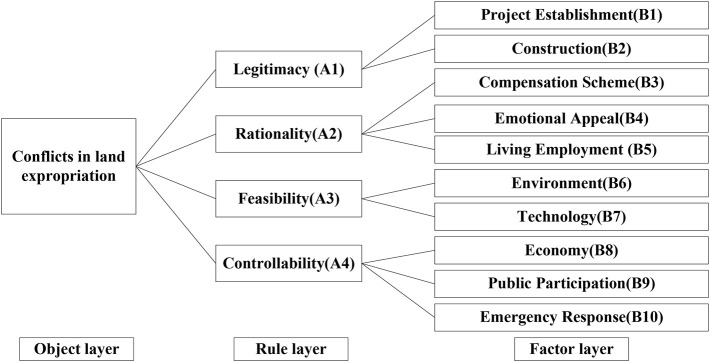
Hierarchical structure for risk assessment of conflicts in land expropriation.

A set of comprehensive and systematic risk evaluation index systems appears to be important in our work. The AHP method is a useful way to analyze complex problems by dividing them into three layers: object layer, rule layer, and factor layer. When we assess risks of some events, the risk is generally regarded as the object layer. Therefore, the first layer is the object layer which is conflicts in land expropriation in this article. The second layer is determined by the official department in China which is proposed by National Development and Reform Commission (NDRC), which state that risk assessment of conflicts in land expropriation should be assessed from the following aspects: legitimacy, rationality, feasibility, and controllability.^1^ The third layer is a further subdivision of the second layer. According to the experts’ responses, 10 major risks points in the factor layer were identified. Based on the information and analysis above, a hierarchical structure for risk assessments of conflicts in land expropriation is established in Figure 4.

#### Judgment Matrix From Proposed Consulting Process

##### Summary of experts’ viewpoints from questionnaire

Based on the hierarchical structure, we designed a questionnaire to evaluate the risk of conflicts in land expropriation as shown in Appendix. We invited six experts to complete the designed questionnaire. Before the experts gave their specific scores, we explained in detail the meaning of each index in the questionnaire and ranking function. What is more, we also introduced two requirements of how to complete questionnaire. These experts have rich experience in conflicts management of land expropriation. The expert scoring has high credibility. Based on the new questionnaire, the authors collected experts’ judgments of each factor in each layer. A summary of the questionnaire responses from the six experts is listed in Appendix. Based on this table, the authors determined an interval number for each influence factor and the times that each score was assigned in the interval.

##### Determination of triangular fuzzy number

In order to illustrate how to determine the triangular fuzzy number and judgment matrix based on the new questionnaire, the authors applied the expert responses of factors A1–A4 in layer 2 to establish the judgment matrix as an example; the specific linguistic variables and the corresponding fuzzy number refer to related publications (Lyu et al., 2020a).

The scores that the six experts assigned to factor A1 ranged from 7 to 9; therefore, A1 was initially assigned with an interval number of 7–9. Regarding the scores of A1, 9 was assigned three times, whereas 7 was assigned only once, and 8 twice. Compared with 7 and 8, the preferred score of A1 was 9. Similarly, A2 = 2–4 (4 was assigned three times, 3 twice, and 2 once). A3 = 3–7 (7 was assigned three times, 4 twice, and 3 once). A4 = 5–7 (7 was assigned three times, 6 was assigned twice, and 5 once). Each element in the judgment matrix can be expressed as the ratio between two interval numbers, such as A1 = (7, 9, 9), A2 = (2, 4, 4), A3 = (3, 7, 7), and A4 = (5, 7, 7).

In order to better illustrate how to determine the triangular fuzzy number, we took the value of A1/A2 as an example. For the value of A1/A2 = (7, 9, 9)/(2, 4, 4), the smallest is 7/4 = 1.75, and the largest is 9/2 = 4.5. Therefore, the value of A1/A2 ranges from 1.75 to 4.5. Based on Satty’s rule, the element in the judgment matrix ranges from 1 to 9 and it should be integer. Therefore, the value of A1/A2 ranges from 2 to 4. What is more, based on the assigned times of different scores, A1 is preferred as 9, A2 is preferred as 4 as an odd number is typically used to express the relative importance in a pairwise comparison (Saaty, 1977). The value of A1/A2 is 2′. Therefore, the authors use triangular fuzzy number 2′ = (2, 2, 4) to represent the value of A1/A2. The value of A2/A1 could also be determined at the reciprocal of A1/A2. Following this method, the pairwise comparison matrix can be expressed using Eq. 4.


(4) $$F_{n\times n}=\left(\begin{array}{ccc} 1 & \text{L} & P_{1\,n}\\ \text{M} & \text{O} & \text{M}\\ P_{n\,1} & \text{L} & 1 \end{array}\right)=\left(\begin{array}{cccc} \frac{A\,1}{A\,1} & \frac{A\,1}{A\,2} & \frac{A\,1}{A\,3} & \frac{A\,1}{A\,4}\\ \frac{A\,2}{A\,1} & \frac{A\,2}{A\,2} & \frac{A\,2}{A\,3} & \frac{A\,2}{A\,4}\\ \frac{A\,3}{A\,1} & \frac{A\,3}{A\,2} & \frac{A\,3}{A\,3} & \frac{A\,3}{A\,4}\\ \frac{A\,4}{A\,1} & \frac{A\,4}{A\,2} & \frac{A\,4}{A\,3} & \frac{A\,4}{A\,5} \end{array}\right)=\left(\begin{array}{cccc} 1 & 2^{\prime } & 1^{\prime } & 1^{\prime }\\ {\frac{1}{2}}^{\prime } & 1 & {\frac{1}{2}}^{\prime } & {\frac{1}{2}}^{\prime }\\ 1^{\prime } & 2^{\prime } & 1 & 1^{\prime }\\ 1^{\prime } & 2^{\prime } & 1^{\prime } & 1 \end{array}\right)$$

Specifically, other triangular fuzzy numbers can be calculated with the same method.

#### Weight Vector From Triangular Fuzzy Analytic Hierarchy Process

Fuzzy analytic hierarchy process is the integration method of qualitative and quantitative methods. The main difference between FAHP and AHP is that FAHP can classify evaluation factors into target level, criterion level, and factor level. However, AHP can only classify evaluation factors into target level and factor level. FAHP effectively avoids the subjectivism of AHP and the one-sided nature of FCE. According to the determined fuzzy numbers and the established triangular fuzzy judgment matrix, the FAHP is applied to determine the eigenvalues and eigenvectors of each judgment matrix (Saaty, 2003). This method refers to the fact that the membership matrix and the corresponding eigenvectors are calculated step by step from the target layer to the criterion layer. Firstly, the membership matrix of the first class index is obtained by the membership matrix and eigenvector of each second-order index. Then, the membership matrix and eigenvector of the first-level index are used to obtain the evaluation set of the first-level index. At last, the whole risk of conflicts in land expropriation can be calculated according to the first-level index.

##### Hierarchical single arrangement

**FIGURE 5:**
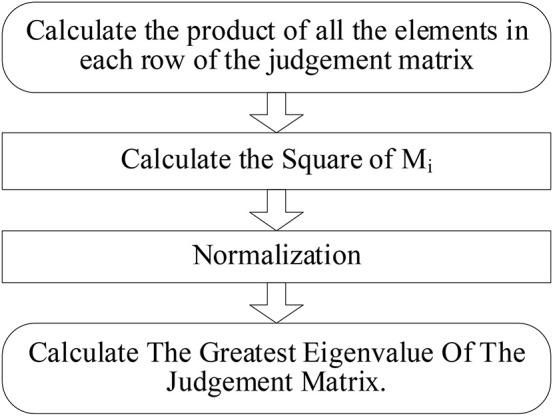
The calculation process of FAHP.

We calculate the eigenvalues and eigenvectors of each judgment matrix using the eigenvalue method. The square root method was adopted to calculate the eigenvalues and eigenvectors of each judgment matrix. And the rule layer will be taken as an example to illustrate how to calculate. The specific steps are reflected in Figure 5.

**Step 1: Calculate the sum of elements in each row of the fuzzy judgment matrix:**


(5) $$M_{i}=\sum_{\text{j}=1}a_{\mathrm{ij}},i=1,2,\cdots$$

In this way, the results of *M*_1_, *M*_2_, *M*_3_ and *M*_4_ can be calculated as $5,\frac{5}{2},5,\text{and}\,5$.

**Step 2: Calculate the square of *M*_*i*_, where “n” represents the rank of matrix:**


$$\bar{W_{i}}=\sqrt[{\text{n}}]{M_{i}}$$

In this way, the results of $\bar{W_{1}}$,$\bar{W_{2}}$, $\bar{W_{3}}$, and$\bar{W_{4}}$ can be calculated as 1.495, 1.257, 1.495, and 1.495.

**Step 3: Normalization, namely:**


(6) $$W_{\text{i}}=\frac{\bar{w_{i}}}{\sum_{\text{j}=1}^{n}\bar{w_{\text{j}}}}$$

And the eigenvectors are expressed as:


(7) $$\text{W}={\left[W_{1},W_{2},W_{3},W_{4}\right]}^{T}$$

(8) $$W_{1}=\frac{\bar{W_{1}}}{\sum_{j=1}^{n}\bar{W_{j}}}=\frac{1.495}{1.495+1.257+1.495+1.495}=0.260$$

Similarly, we can calculate weights of *W*_*2*_, *W*_*3*_, and *W*_*4*_ as *W*_2_ = 0.220, *W*_3_ = 0.260, and *W*_4_ = 0.260.

The set of weights is *W* = [0.260, 0.220, 0.260,0.260].

**Step 4: Calculate the greatest eigenvalue of the judgment matrix:**


(9) $$\lambda_{\max}=\sum_{i=1}^{n}\frac{{\mathrm{AW}}_{i}}{{\mathrm{nW}}_{i}}$$

A is the priority judgment matrix, and *W*_*i*_ represents the corresponding eigenvector. *A* = [a_*ij*_]_*n*×*n*_.

a_*ij*_ is represented by elements in the “*i*” row and “*j*” column of the judgment matrix. 1≤*i*≤*n*, 1≤*j*≤*n*.


(10) $$\text{AW}=\left[\begin{array}{cccc} 1 & 2^{\prime } & 1^{\prime } & 1^{\prime }\\ {\frac{1}{2}}^{\prime } & 1 & {\frac{1}{2}}^{\prime } & {\frac{1}{2}}^{\prime }\\ 1^{\prime } & 2^{\prime } & 1 & 1^{\prime }\\ 1^{\prime } & 2^{\prime } & 1^{\prime } & 1 \end{array}\right]\times \left[\begin{array}{c} \begin{array}{c} 0.260\\ 0.220 \end{array}\\ \begin{array}{c} 0.260\\ 0.260 \end{array} \end{array}\right]=\left[\begin{array}{c} \begin{array}{c} 1.219\\ 0.609 \end{array}\\ \begin{array}{c} 1.219\\ 1.219 \end{array} \end{array}\right]$$

The greatest eigenvalue is expressed as:


(11) $$\lambda_{m\,a\,x}=\sum_{i=1}^{n}\frac{{\left(A\,W\right)}_{i}}{n\,W_{i}}=\frac{{\left(A\,W\right)}_{1}}{4\,W_{1}}+\frac{{\left(A\,W\right)}_{2}}{4\,W_{2}}+\frac{{\left(A\,W\right)}_{3}}{4\,W_{3}}+\frac{{\left(A\,W\right)}_{4}}{4\,W_{4}}$$

We can calculate the eigenvalue as: $\lambda_{m\,a\,x}=\frac{1.219}{4\times 0.260}+\frac{0.502}{4\times 0.220}+\frac{1.219}{4\times 0.260}+\frac{1.219}{4\times 0.260}=4.207$.

##### The consistency test

In order to verify the consistency of FAHP, random consistency indicators of section numbers in the judging matrix are given to solve this problem (Hamidi et al., 2010). The consistency check is useful for analyzing the harmonization of the experts’ judgment, researching the relationship of each index. In particular, the paradox such as A is more important than B, B is more important than C, but C is more important than A can be avoided. The meaning of “CR” represents the consistency ratio.


(12) $$\text{CR}=\frac{\text{CI}}{\text{RI}}$$

When *CR* < 0.1, the consistency of the judgment matrix is within an acceptable range. When "*CR*≥0.1", the consistency of the judgment matrix is not an acceptable range, and the judgment matrix needs to be adjustment. “CI” means the consistency indicators.


(13) $$\text{CI}=\frac{\lambda_{\text{max}}-n}{n-1}$$

The meaning of RI is the random consistency matrix; it is related to the order of the judgment matrix. When the order of the judgment matrix is greater, the number value of the “RI” is greater. For details see attached Table 1.

**TABLE 1:** The mean random consistency index.

	**1**	**2**	**3**	**4**	**5**	**6**	**7**	**8**	**9**	**10**
RI	0	0	0.52	0.89	1.12	1.26	1.36	1.41	1.46	1.49

Take the rule layer (one class index), for example, the consistency check is used to conduct a comprehensive analysis. The eigenvalues can be calculated.


$$\lambda_{\max}=4.207,n=4,R\,I=0.89,\text{CI}=\frac{\lambda_{m\,a\,x}-n}{n-1}=\frac{4.207-4}{4-1}=0.069,\mathrm{CR}=\frac{0.069}{0.89}=0.078<0.1.$$

The above indexes are all better than the standards, and the judgment matrix is constructed through the consistency test. In this way, the factor layer (second class index) of the judgment matrix can be tested, and all of the two class indexes passed through the consistency test. The value of the rationality judgment matrix is 0.069 < 0.1; the value of the feasibility judgment matrix is 0.078 < 0.1. It is noteworthy that the judgment matrix of legality and controllability only have two numbers, and it corresponds to the consistent standard. There is no need to do the consistency for the judgment matrix of legality and controllability.

##### Hierarchical population ordering

Specific steps for calculating the index of the rule layer is as follows: firstly, each column of priority judgment matrix A is normalized to get matrix A1; then, vector A2 is obtained by adding the elements of each row of matrix A1. At last, the weight vector A4 of the first level index (criterion level) is obtained by normalizing each row element in vector A3. Similarly, the same calculation is made for the index layer (secondary index). Finally, the weights and the mean random consistency of each index layer are shown in Table 2.

**TABLE 2:** Weights of factors in layers 1–2.

**Layer 1**	**Layer 2**	**Synthesized weights**	**The mean random consistency**
Legitimacy (0.260)	Legitimacy of project establishment (0.75)	0.195	CI = 0, λ_max_ = 2
	Legitimacy of construction (0.25)	0.065	RI = 0
Rationality (0.220)	Rationality of compensation scheme (0.615)	0.135	CI = 0.037
	Rationality of emotional appeal (0.117)	0.026	λ_max_ = 3.074
	Rationality of living employment (0.268)	0.059	RI = 0.52, CR = 0.071
Feasibility (0.260)	Feasibility of economy (0.637)	0.166	CI = 0.037, RI = 0.52
	Feasibility of technology (0.105)	0.027	λ_max_ = 3.039
	Feasibility of environment (0.258)	0.060	CR = 0.037
Controllability (0.260)	Controllability of public participation (0.25)	0.065	CI = 0, λ_max_ = 2
	Controllability of emergency response (0.75)	0.195	RI = 0

According to the triangular fuzzy judgment and fuzzy number, we could calculate the synthesized weights of each layer. Table 1 reflects the weights of factors in each layer and the mean random consistency. Which reflects the ranking risks of conflicts in land expropriation in SY city. The shape of fuzzy values in the AHP model is showed in Figure 5.

**FIGURE 6:**
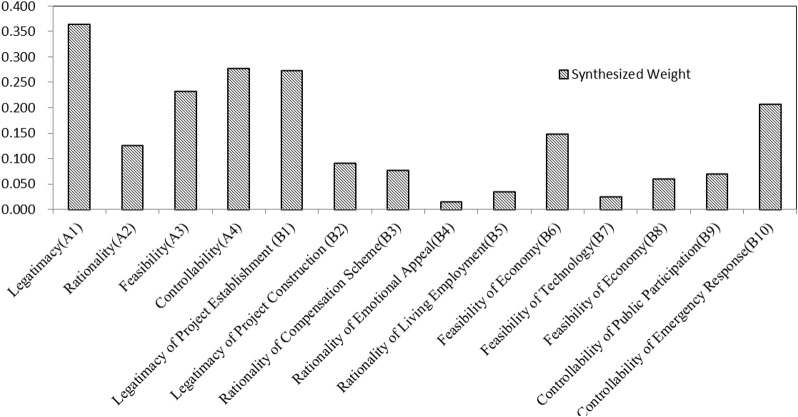
Synthesized weights for ranking risks in conflicts of land expropriation.

Figure 6 indicates the synthesized weights for ranking the risk factors with the triangular FAHP based on the conflicts of land expropriation that occurred in SY. As indicated in Figure 4, at the first layer, the legitimacy is the most critical factor that may pose the worst conflicts risk to land expropriation in SY city which has the largest weights, followed by controllability (A4), feasibility (A3), and rationality (A2). Therefore, legitimacy of land expropriation is most likely to cause conflicts risks. At the second layer, the legitimacy of project establishment (B1) is the most significant risk in land expropriation, which is much more important than the legitimacy of project construction, followed by controllability of emergency response (B10). Generally speaking, the ranking results show that more attention should be focused on the legitimacy of project establishment, controllability of emergency response, and feasibility of economy for most of the land expropriation projects in SY.

### Conflicts Risk Assessment of Specific Land Expropriation Using Fuzzy Comprehensive Evaluation

#### Steps of Conflicts Risk Assessment for Specific Land Expropriation

In order to demonstrate the application of the proposed risk assessment methods, one specific land expropriation project was selected as a case study.

##### Establish the factor set of comprehensive evaluation

First, this part will establish the index set of the first class index U, and the second class index

*U*_i_(**i** = **1**,** 2**,** 3**,** 4**) is based on the context. The index set of the rule layer is:


U={Leg⁢itimac⁢y,⁢Rationality,⁢Feasibility,⁢Controllability}

The index set of the factor layer is as following:


$$U_{1}=\left\{\mathrm{Project}\,\,\mathrm{Establishment},\,\mathrm{Construction}\right\}$$

$$U_{2}=\{\mathrm{Compensation}\mathrm{Scheme},\mathrm{Emotional}\mathrm{Appeal},\mathrm{Living}\,\mathrm{Employment}\}$$

$$U_{3}=\left\{\mathrm{Economy},\,\mathrm{Technology},\,\mathrm{Environment}\right\}$$

$$U_{4}=\left\{\mathrm{Public}\,\,\mathrm{participation},\,\mathrm{emergency}\,\,\mathrm{response}\right\}$$

##### Establish the evaluation set of comprehensive evaluation

According to rules issued last year by the NDRC in China, the risk level of the land must be divided into three levels, for details see Table 3.

**TABLE 3:** The risk assessment grading of land conflict.

**V_*k*_**	**Risk rank**	**Scalar implicates**
High risk	Three	Majority of community populace are opposed to the development plan which may lead to land conflict to a large extent.
Medium risk	Two	Some of the community populace are opposed to the development plan, and parts of them are emotionally or mentally disturbed.
Low risk	One	Most of the community populace can understand and support the land exploitation, and contradictions can be prevented and resolved according to the effective work.

And therefore, the evaluation remarks can be listed as the following set:

##### Establish the fuzzy evaluation matrix

While confirming the methods of performance evaluation, this article introduces an analytical hierarchy process and FCE. In this way, the qualitative evaluation index is transformed into a quantitative evaluation index. Therefore, this article needs to establish a judgment matrix.

In the empirical study, we provide the basic situation of this project and an Analysis of Questionnaire Survey of the surrounding residents. Then we use scores taken from experts to evaluate the risk factors, and finally utilize the FCE method to calculate the various kinds of risk. A total of 15 experts and scholars were invited to evaluate and score in this study. The specific scoring results are shown in Table 4.

**TABLE 4:** The evaluation criteria system and marks.

**First class index**	**Second class index**	**Low risk**	**Medium risk**	**High risk**
Legitimacy (0.260)	Project establishment (0.750)	9	4	2
	Construction (0.250)	7	5	3
Rationality (0.220)	Compensation scheme (0.608)	9	5	1
	Emotional appeal (0.120)	8	7	0
	Living employment (0.272)	9	5	1
Feasibility (0.260)	Economy (0.633)	6	9	0
	Technology (0.106)	6	5	4
	Environment (0.260)	8	3	4
Controllability (0.260)	Public participation (0.250)	5	8	2
	Emergency response (0.750)	5	9	1

*R*_*i*_(*i* = 1, 2, 3, 4) is the judgment matrixes of the second class index, R is the judgment matrixes of the first class index. And four judgment matrixes of the second class index can be calculated through the expert evaluation method. The sources of the applied equations were based on the previous literature of FCE (Liao et al., 2018; Pu et al., 2018; Lyu et al., 2019).


(14) $$\begin{array}{cc} \text{R}\,1=\left[\begin{array}{cc} 0.600 & \begin{array}{cc} 0.270 & 0.133 \end{array}\\ 0.467 & \begin{array}{cc} 0.333 & 0.200 \end{array} \end{array}\right] & \text{R}\,2=\left[\begin{array}{ccc} 0.600 & 0.333 & 0.067\\ 0.533 & 0.467 & 0\\ 0.600 & 0.333 & 0.067 \end{array}\right]\\ \text{R}\,3=\left[\begin{array}{ccc} 0.600 & 0.400 & 0\\ 0.400 & 0.333 & 0.067\\ 0.533 & 0.200 & 0.267 \end{array}\right] & \text{R}\,4=\left[\begin{array}{cc} 0.333 & \begin{array}{cc} 0.533 & 0.133 \end{array}\\ 0.333 & \begin{array}{cc} 0.6 & 0.607 \end{array} \end{array}\right] \end{array}$$

*Wi*(*i* = 1, 2, 3, 4) is the weight vector of the factor layer (second class index) which includes the legality indictors, rationality indictors, feasibility indictors, and controllability indictors.


(15) $$\begin{array}{cc} W_{1}=\left[\begin{array}{cc} 0.75 & 0.25 \end{array}\right] & W_{2}=\left[\begin{array}{ccc} 0.608 & 0.120 & 0.272 \end{array}\right]\\ W_{3}=\left[\begin{array}{ccc} 0.633 & 0.107 & 0.260 \end{array}\right] & W_{4}=\left[\begin{array}{cc} 0.25 & 0.75 \end{array}\right] \end{array}$$

*Bi*(*i* = 1, 2, 3, 4) is the evaluation vector of the factor layer (second class index):


(16) $$\begin{array}{c} B\,1=\text{W}\,1\times \text{R}\,1=\left[\begin{array}{ccc} 0.567 & 0.283 & 0.150 \end{array}\right]\\ \text{B}\,2=\text{W}\,2\times \text{R}\,2=\left[\begin{array}{ccc} 0.592 & 0.349 & 0.059 \end{array}\right]\\ \text{B}\,3=\text{W}\,3\times \text{R}\,3=\left[\begin{array}{ccc} 0.434 & 0.469 & 0.097 \end{array}\right]\\ \text{B}\,4=\text{W}\,4\times \text{R}\,4=\left[\begin{array}{ccc} 0.333 & 0.583 & 0.084 \end{array}\right] \end{array}$$

Meanwhile, the evaluation matrix *R* of the rule layer (first class index) is represented as the following:


(17) $$R=\left[\begin{array}{c} \begin{array}{c} B\,1\\ B\,2 \end{array}\\ \begin{array}{c} B\,3\\ B\,4 \end{array} \end{array}\right]=\left[\begin{array}{c} \begin{array}{ccc} 0.5670.2830.150 & & \end{array}\\ \begin{array}{ccc} 0.5920.3490.059 & & \end{array}\\ \begin{array}{ccc} 0.4340.4690.097 & & \end{array}\\ \begin{array}{ccc} 0.3330.5830.084 & & \end{array} \end{array}\right]$$

Therefore, the evaluation vector of the rule layer (one class index) can be calculated as follows:


(18) $$B=W\times R=\left[\begin{array}{ccc} 0.477 & 0.605 & 0.133 \end{array}\right]$$

#### Results

According to the maximum subordination principle, we can see from Figure 6 that the membership degree of different risk level shows that the maximum membership degree is 0.605 which belongs to the medium risk level. It shows that the land conflict risk of the project is within the scope of control, and the project can continue safely. The maximum membership degree of the legitimacy index score is 0.567, which belongs to the low risk level; the maximum membership degree of the rationality score is 0.592, which is at a low risk level. The maximum membership degree of the feasibility score is 0.467, which belongs to the medium risk level. The maximum membership degree of the controllability score is 0.583, which is also at medium risk. Most noteworthy, as the feasibility and controllability of the secondary indicators are in the middle risk level, it is suggested that the government should troubleshoot risk points from this land development.

##### Legitimacy

The legitimacy rating of this project is at low risk. The investigation found that the project strictly complied with the approval procedure for project establishment. It conforms to the local industrial development plan. What is more, the relevant qualifications of construction personnel have been examined according to law, so the legitimacy is at low risk.

##### Rationality

The rationality evaluation grade of this project is at low risk. According to investigations, this article found three things of note. Firstly, in the aspects of the compensation scheme, this project implements monetization of one-time compensation and purchase of commodity housing stock in the county market. The compensation scheme is reasonable. Secondly, in the aspects of emotional appeal, 97% of the respondents believed that it had no impact on local culture, customs, and religion after the project was completed. However, 2% of the respondents believed that it had some influence on customs, and 1% of the respondents believed that it had some influence on culture. Thirdly, in the aspects of living employment, it will not have a great impact on the employment of Aboriginal residents because the demolished project does not have a commercial network of villages in the city. However, considering that the expropriated and demolished residents need to establish new neighborhood relations after demolition, there will be short-term maladjustment.

##### Feasibility

The feasibility evaluation grade of this project is at medium risk. Therefore, further analysis of economic, technological, and environmental feasibility reveals the following risk sources. In terms of economic feasibility, it is found that similar plots have not been investigated. In terms of environmental feasibility, a small amount of dust will be produced in the process of land expropriation, and a large amount of construction waste will affect the living environment of the surrounding residents. In terms of technical feasibility, the lives of the surrounding residents will be disturbed due to technical constraints, excavators, loaders, automobile transportation, and other noise and vibrations.

##### Controllability

The controllability evaluation grade of the urban village project is at medium risk. The risk sources are identified further according to the two indicators of public participation and emergency response. The following problems were found. In terms of public participation, after hearing of the land requisition and demolition plan, about four residents disagreed with the plan, however, relevant departments did not follow-up on the solution As for emergency response, there were no negative reports from mainstream news media, such as online newspapers. However, relevant authorities did not prepare measures to network public opinion.

## Discussion

The FAHP-FCE was successfully applied to evaluate the risk degree of land expropriation in SY. The major findings are summarized as follows: (1) a set of risk evaluation indexes system was established based on experts’ opinions using the proposed method. (2) A risk analysis of the SY land expropriation illustrated the efficiency and validity of the FAHP-FCE in the risk assessment of land expropriation. The results of risk assessment for specific land expropriation projects using the proposed method show that the overall risk of specific land expropriation projects was at a low level; however, the feasibility and controllability of the second layer is at the medium level which need to be paid more attention to. (3) It can better prevent conflicts risk in advance based on the evaluation results of land expropriation. The risk degree of land expropriation can be evaluated in advance using FAHP-FCE, and some specific risk points can be removed and qualify the land expropriation system. The contribution of this article shows that:

1. The application of FAHP into risk assessment in land expropriation is effective, which enlarges the application scope of FAHP. Although FAHP has a wide application in risk assessment for many projects, the applicability of FAHP in risk assessments in conflicts in land expropriation is still unclear. The system can be determined, and also ranks risks from the perspective of the system as a whole.
2. The FAHP-FCE methodology enriches management means of conflicts in land expropriation. It is helpful for relevant departments to establish a strict management system of work safety for conflicts in land exploration based on the risk assessment results. The FAHP assessment method uses a triangular fuzzy number instead of a crisp number, which overcomes the one-sided deficit of the original AHP method. It overcomes the shortcomings of the simple weighted average method, and it makes up for the deficiency of the traditional AHP and improves the fault tolerance of the model. At the same time, it breaks through the limitations of traditional risk assessment methods and integrates the probabilistic risk assessment method and risk FCE method.

There are also some limitations in this article, because Saaty’s AHP produces rank reversal, a new procedure was proposed based on a simple algebraic system of equations, called “Alpha-Discounting Method for Multi-Criteria Decision Making” (Smarandache, 2015), which is considered for multi-criteria decision-making as a future study.

## Data Availability Statement

The original contributions presented in the study are included in the article/supplementary material, further inquiries can be directed to the corresponding author/s.

## Ethics Statement

Ethical review and approval was not required as per local legislation and institutional requirements because the protocol involved the invitation of six experts to complete the designed questionnaire using the method of FCE-AHP. Written informed consent was not required for the study on human participants in accordance with the local legislation and institutional requirements.

## Author Contributions

GP: acquization of original data, methodology, and funding acquisition. LH: writing – review and editing, methodology, and manuscript revision. ZL: reference management and manuscript revision critically. YG: data calculation and writing. JY: conceptualization and methodology. XJ: software and formal analysis. All authors contributed to the article and approved the submitted version.

## Conflict of Interest

YG was employed by the company China Ordnance Materials Huabei Co., Ltd. The remaining authors declare that the research was conducted in the absence of any commercial or financial relationships that could be construed as a potential conflict of interest.

## Publisher’s Note

All claims expressed in this article are solely those of the authors and do not necessarily represent those of their affiliated organizations, or those of the publisher, the editors and the reviewers. Any product that may be evaluated in this article, or claim that may be made by its manufacturer, is not guaranteed or endorsed by the publisher.

## Footnotes

1
https://www.ndrc.gov.cn/fggz/gdzctz/tzfg/201907/t20190717_1197572.html

## Appendix

**TABLE A1:** Questionnaire for risk assessment of conflicts in land expropriation.

**Layer**	**Factor**	**Influence of the factors on the risk of conflicts in land expropriation**								
		**1**	**2**	**3**	**4**	**5**	**6**	**7**	**8**	**9**
Layer 1	Legitimacy							I	II	III
	Rationality		I	II	III					
	Feasibility			I	II			III		
	Controllability					I	II	III		
Layer 2	Project establishment							I	II	III
	Construction			III	II	I				
	Compensation scheme						I	II	III	
	Emotional appeal		III	I	I	I				
	Living employment			III	II	I				
	Environment							I	II	III
	Technology		III	I	II					
	Economy		II	III	I	I				
	Public participation			III	II	I	I			
	Emergency response				I	II	III			

## References

[B1] Abdo H. Flaus J. M. (2016). Uncertainty quantification in dynamic system risk assessment: a new approach with randomness and fuzzy theory. *Int. J. Prod. Res.* 54 5862–5885. 10.1080/00207543.2016.1184348

[B2] Bao H. J. Wu X. H. Wang H. W. Li Q. X. Peng Y. Lu S. B. (2019). Conflicts Induced by different responses to land expropriation among the farmers involved during urbanization in China. *JASSS* 22:7.

[B3] Cao Y. G. Dallimer M. Stringer L. C. Bai Z. K. Siu Y. L. (2018). Land expropriation compensation among multiple stakeholders in a mining area: explaining “skeleton house” compensation. *Land Use Policy* 74 97–110. 10.1016/j.landusepol.2017.09.003

[B4] Chen Y. Shu L. C. Burbey T. J. (2014). An integrated risk assessment model of township-scaled land subsidence based on an evidential reasoning algorithm and fuzzy set theory. *Risk Anal.* 34 656–669. 10.1111/risa.12182 24593262

[B5] Faghih-Roohi S. Xie M. Ng K. M. (2014). Accident risk assessment in marine transportation via Markov modelling and Markov chain Monte Carlo simulation. *Ocean Eng.* 91 363–370. 10.1016/j.oceaneng.2014.09.029

[B6] Feng L. Zhu X. D. Sun X. (2014). Assessing coastal reclamation suitability based on a fuzzy-AHP comprehensive evaluation framework: a case study of lianyungang, China. *Mar. Pollut. Bull.* 89 102–111. 10.1016/j.marpolbul.2014.10.029 25455377

[B7] Geng Y. F. Zheng X. Wang Z. L. Wang Z. W. (2020). Flood risk assessment in Quzhou City (China) using a coupled hydrodynamic model and fuzzy comprehensive evaluation (FCE). *Nat. Hazards* 100 133–149. 10.1007/s11069-019-03803-0

[B8] Hamidi J. K. Shahriar K. Rezai B. Rostami J. Bejari H. (2010). Risk assessment based selection of rock TBM for adverse geological conditions using Fuzzy-AHP. *Bull. Eng. Geol. Environ.* 69 523–532. 10.1007/s10064-009-0260-8

[B9] He Z. Y. Asami Y. (2014). How do landowners price their lands during land expropriation and the motives behind it: an explanation from a WTA/WTP experiment in central Beijing. *Urban Stud.* 51 412–427. 10.1177/0042098013492227

[B10] Hui E. C. M. Bao H. J. (2013). The logic behind conflicts in land acquisitions in contemporary China: a framework based upon game theory. *Land Use Policy* 30 373–380. 10.1016/j.landusepol.2012.04.001

[B11] Jiang W. G. Deng L. Chen L. Y. Wu J. J. Li J. (2009). Risk assessment and validation of flood disaster based on fuzzy mathematics. *Prog. Nat. Sci. Mater. Int.* 19 1419–1425. 10.1016/j.pnsc.2008.12.010

[B12] Lee S. (2015). Determination of priority weights under multiattribute decision-making situations: AHP versus fuzzy AHP. *J. Constr. Eng. Manag.* 141:05014015. 10.1061/(asce)co.1943-7862.0000897 29515898

[B13] Li C. X. Xi Z. L. (2019). Social stability risk assessment of land expropriation: lessons from the Chinese case. *Int. J. Environ. Res. Public Health* 16:3952. 10.3390/ijerph16203952 31627312 PMC6843856

[B14] Li F. W. Phoon K. K. Du X. L. Zhang M. J. (2013). Improved AHP method and its application in risk identification. *J. Constr. Eng. Manag.* 139 312–320. 10.1061/(asce)co.1943-7862.0000605 29515898

[B15] Liao Y. Q. Yu G. H. Liao Y. Jiang L. Liu X. Z. (2018). Environmental conflict risk assessment based on AHP-FCE: a case of jiuhua waste incineration power plant project. *Sustainability* 10:4095. 10.3390/su10114095

[B16] Liu L. Zhou J. Z. An X. L. Zhang Y. C. Yang L. (2010). Using fuzzy theory and information entropy for water quality assessment in Three Gorges region, China. *Expert Syst. Appl.* 37 2517–2521. 10.1016/j.eswa.2009.08.004

[B17] Lyu H. M. Shen S. L. Yang J. Zhou A. N. (2020a). Risk Assessment of earthquake-triggered geohazards surrounding Wenchuan. *China. Nat. Hazards Rev.* 21:05020007.

[B18] Lyu H. M. Shen S. L. Zhou A. N. Yang J. (2020b). Risk assessment of mega-city infrastructures related to land subsidence using improved trapezoidal FAHP. *Sci. Total Environ.* 717:135310. 10.1016/j.scitotenv.2019.135310 31839300

[B19] Lyu H. M. Shen S. L. Zhou A. N. Zhou W. H. (2019). Data in flood risk assessment of metro systems in a subsiding environment using the interval FAHP-FCA approach. *Data Brief* 26:104468. 10.1016/j.dib.2019.104468 PMC6811962 31667235

[B20] Ma X. L. Dai M. L. Fan D. X. F. (2020). Land expropriation in tourism development: residents’ attitudinal change and its influencing mechanism. *Tour. Manag.* 76:103957.

[B21] Mathur S. (2013). Use of land pooling and reconstitution for urban development: experiences from Gujarat. *India. Habitat Int.* 38 199–206.

[B22] Pu H. X. Luo K. L. Zhang S. X. (2018). Risk assessment model for different foodstuff drying methods via AHP-FCE method: a case study of “coal-burning” fluorosis area of Yunan and Guizhou Province. *China. Food Chem.* 263 74–80. 29784330 10.1016/j.foodchem.2018.04.123

[B23] Qiu G. Q. Huang S. Zhu L. L. Su X. H. Chen Y. (2015). Risk assessment of multi-state bayesian network in an oil gathering and transferring system. *Press. Vessel Technol.* 130 1514–1523. 10.1016/j.proeng.2015.12.320

[B24] Saaty T. L. (1977). Scaling method for priorities in hierarchical structures. *J. Math. Psychol.* 15 234–281. 10.1016/0022-2496(77)90033-5

[B25] Saaty T. L. (2003). Decision-making with the AHP: why is the principal eigenvector necessary. *Eur. J. Oper. Res.* 145 85–91. 10.1016/S0377-2217(02)00227-8

[B26] Smarandache F. (2010). “α-Discounting method for multi-criteria decision making (α-D MCDM),” in *Proceedings of Fusion 2010 International Conference*, Scotland 10.1109/ICIF.2010.5712044

[B27] Smarandache F. (2013a). “Interval alpha-discounting method for MCDC,” in *Proceedings of the Annual Symposium of the Institute of Solid Mechanics and Session of the Commission of Acoustics (The 24th SISOM)*, (Gallup, NM: University of New Mexico).

[B28] Smarandache F. (2013b). “Three non-linear α-discounting MCDM-method examples,” in *Proceedings of the 2013 International Conference on Advanced Mechatronic Systems (ICAMechS 2013)*, (Gallup, NM: University of New Mexico). 10.1109/ICAMechS.2013.6681772

[B29] Smarandache F. (2015). *α-Discounting Method for Multi-Criteria Decision Making (α-D MCDM).* Columbus: Romania & Educational Publisher. 10.2139/ssrn.2720888

[B30] Sun B. D. Tang J. C. Yu D. H. Song Z. W. Wang P. G. (2019). Ecosystem health assessment: a PSR analysis combining AHP and FCE methods for Jiaozhou Bay, China. *Ocean Coastal Manag.* 168 41–50. 10.1016/j.ocecoaman.2018.10.026

[B31] Tagliarino N. K. Bununu Y. A. Micheal M. O. De Maria M. Olusanmi A. (2018). Compensation for expropriated community farmland in nigeria: an in-depth analysis of the laws and practices related to land expropriation for the lekki free trade zone in lagos. *Land* 7:23. 10.3390/land7010023

[B32] Taylan O. Bafail A. O. Abdulaal R. M. S. Kabli M. R. (2014). Construction projects selection and risk assessment by fuzzy AHP and fuzzy TOPSIS methodologies. *Appl. Soft Comput.* 17 105–116. 10.1016/j.asoc.2014.01.003

[B33] Wang L. J. (2019). Research on human resource performance and decision-making evaluation based on fuzzy mathematics and clustering model. *J. Intell. Fuzzy Syst.* 37 171–184. 10.3233/JIFS-179075

[B34] Zhou Z. Q. Li S. C. Li L. P. Shi S. S. Xu Z. H. (2015). An optimal classification method for risk assessment of water inrush in karst tunnels based on grey system theory. *Geomech. Eng.* 8 631–647. 10.12989/gae.2015.8.5.631

[B35] Zou Q. Zhou J. Z. Zhou C. Song L. X. Guo J. (2013). Comprehensive flood risk assessment based on set pair analysis-variable fuzzy sets model and fuzzy AHP. *Stoch. Environ. Res. Risk Assess.* 27 525–546. 10.1007/s00477-012-0598-5

